# Supplementary material for: Maternal Sociodemographic Factors and Antenatal Stress
Source: Int J Environ Res Public Health. 2021 Jun 25;18(13):6812. doi: 10.3390/ijerph18136812 (PMC8297233; doi:10.3390/ijerph18136812)
Supplement: Supplementary file 1 [file ijerph-18-06812-s001.zip › ijerph-1231453-supplementary.pdf]

Table S1. Extract from the Sociodemographic and Clinical Questionnaire

Study ID:	
Information Collected by:	
Date:	
Contact Information	
Cell phone No.	
Area of residence/community	
Alternate contact Cell phone No.	
Alternate contact relationship to study participant:	

Demographic Information

1. Mother born in Kenya	Kenya<br>□ Yes<br>□ No<br>If no, how long has she lived in Kenya…Years…months
2. Mother's Age	____________________Years
3. Mother's ethnic background	□ Luo<br>□ Kuria<br>□ Kisii<br>□ Mixed/Other:<br>__________________________
4. Mother's highest education level	□ No Education<br>□ Primary level<br>□ Secondary level<br>□ Technical college/Trade<br>□ Professional college<br>□ University Bachelor's degree<br>□ University [Master's PhD]

4. Mother's Occupation	□ House wife<br>□ Self employed<br>□ Government job<br>□ Private job<br>□ Other, specify: ____________________
5. Mother's income per month (net)	□ Less than KES 5,000<br>□ KES 5,001– 10,000<br>□ KES 10,001 – 20,000<br>□ KES 20,001 – 30,000<br>□ KES 30,001 – 40,000<br>□ KES 40,001 – 50,000<br>□ above KES 50,000<br>KES = Kenyan Shillings
6. Baby's Father born in	Kenya<br>□ Yes<br>□ No<br><br>If no, how long has he lived in Kenya _____ Years<br>months
7. Baby's Father Age	_________Years
8. Baby's Father ethnic background	□ Luo<br>□ Kuria<br>□ Kisii<br>□ Mixed/Other
9. Baby's Father highest education level	□ No Education<br>□ Primary level<br>□ Secondary level<br>□ Technical college/Trade<br>□ Professional college<br>□ University Bachelor's degree<br>□ University [Master's PhD]
10. Baby's Father Occupation	□ Unemployed<br>□ Self employed<br>□ Government job<br>□ Private job<br>□ Other, specify: ____________________
11. Baby's Father income per month	□ Less than KES 5,000<br>□ KES 5,001 – 10,000

	□ KES 10,001 – 20,000<br>□ KES 20,001 – 30,000<br>□ KES 30,001 – 40,000<br>□ KES 40,001 – 50,000<br>□ More than KES 50,000<br><br>KES = Kenyan Shillings
12. Combined Family Income per Month (net)	□ Less than KES 10,000<br>□ KES 10,001 – 20,000<br>□ KES 20,001 – 30,000<br>□ KES 30,001 – 40,000<br>□ KES 40,001 – 50,000<br>□ More than KES 50,000<br><br>KES = Kenyan Shillings
13. Marital Status	□ Single<br>□ Cohabitation<br>□ Traditional Marriage<br>□ Civil Marriage<br>□ Religious Marriage
14. As far as you are aware, are you in a polygamous relationship?	□ Yes<br>□ No
15. Number of families in the house:	□ Two or more (e.g. brother-in-law other relatives e.g. uncles, aunts<br>□ No (husband and children only)
16. How many people live with you in the house?	____________ People<br>[Insert number]

	□ pill<br>□ 3 monthly depo injection<br>□ Copper IUD<br>□ Standard Days Method<br>□ Tubal ligation<br>□ other, specify
24. Gravid:	□ Primiparous (i.e., first pregnancy)<br>□ Multiparous (i.e., given birth ≥ two times)<br><br>If multiparous:<br>a) Total number of children: ___<br>b) Previous male<br>□ Yes<br>□ No<br><br>Previous Preterm birth (<37 weeks)<br>□ Yes<br>□ No
25. When was your last baby born?	_____ Date ____month___Year<br>Calculate Interpregnancy interval<br>_____________Months
26. Did you have any significant medical problem/s prior to pregnancy? (Check all that apply)	□ Malaria<br>□ HIV<br>□ Diabetes<br>□ Asthma<br>□ Hypertension<br>□ History of depression<br>□ Other
27. Have you had and/or<br>been treated for infections during pregnancy?	□ Yes<br>□ No<br>If yes, which (Circle all that apply)<br><br>▯ Vaginal infections<br>▯ Urinary tract infections<br>▯ Dental related infections<br>▯ Sexually transmitted infections<br>▯ Respiratory infections (e.g. pneumonia, bronchitis)<br>▯ Fever episodes<br>▯ Flu/Cold like symptoms

36. Since becoming pregnant, have you at any point been subjected to violence or violent behavior from your partner?	☐ Yes<br>☐ No<br><br>If yes, what type of violence were you exposed to?<br><br>☐Intense verbal abuse<br>☐Slapping and kicking<br>☐Major physical injuries requiring medical attention<br>☐Forced sexual activity<br>☐Evicted from homestead to go and live with other relatives<br>☐Other, specify
38. How do you cope when you feel "stressed"?	☐I develop my own inner strength to cope<br>☐I mostly share my problems with my close friends and family (e.g. mother, sister, mother-in-law, sister-in-law etc.)<br>☐I go to church and attend religious meetings<br>☐I go to live with family or friends who are welcoming<br>☐Other, specify

Table S2. The Perceived Stress Scale (PSS-10)

The questions in this scale ask you about your feelings and thoughts during the last month. **In each case, you will be asked to indicate by circling *how often* you felt or thought a certain way.**

Name ______________________________ Date __________

Age ______ Gender (*Circle*): **M F** Other ______________________

0 = Never 1 = Almost Never 2 = Sometimes 3 = Fairly Often 4 = Very Often

1. In the last month, how often have you been upset because of something that happened unexpectedly?	0	1	2	3	4
2. In the last month, how often have you felt that you were unable to control the important things in your life?	0	1	2	3	4
3. In the last month, how often have you felt nervous and "stressed"?	0	1	2	3	4
4. In the last month, how often have you felt confident about your ability to handle your personal problems?	0	1	2	3	4
5. In the last month, how often have you felt that things were going your way?	0	1	2	3	4
6. In the last month, how often have you found that you could not cope with all the things that you had to do?	0	1	2	3	4
7. In the last month, how often have you been able to control irritations in your life?	0	1	2	3	4
8. In the last month, how often have you felt that you were on top of things?	0	1	2	3	4
9. In the last month, how often have you been angered because of things that were outside of your control?	0	1	2	3	4
10. In the last month, how often have you felt difficulties were piling up so high that you could not overcome them?	0	1	2	3	4

Table S3. Prenatal Medical Risk Index

Use 0 if not present, 1 if present

Unusual features of pregnancy

1. ______ diabetes (current or past)
2. ______ heart disease (current or past)
3. ______ polyhydramnios
4. ______ communicable disease
5. ______ Rh negative

Gynecological and obstetric history

6. ______ 5 or more past deliveries
7. ______ previous fetal demise
8. ______ previous stillbirth
9. ______ previous preterm delivery
10. ______ previous 2nd trimester spontaneous abortion
11. ______ 3 or more previous 1st trimester spontaneous abortions
12. ______ 2 or more therapeutic abortions

Complications of past pregnancies

13. ______ hemorrhage
14. ______ placenta previa
15. ______ pregnancy induced hypertension
16. ______ pre-eclampsia (toxemia)
17. ______ gestational diabetes

Family history

18. ______ diabetes

Patient history (can be identified in current pregnancy as long as not counted twice)

19. _____ hypertension
20. _____ pulmonary disease (or current asthma)
21. _____ genito-urinary infections
22. _____ renal disease
23. _____ epilepsy
24. _____ uterine surgery
25. _____ uterine or cervical abnormality
26. _____ herpes

Current pregnancy complaints

27. _____ anemia
28. _____ flu syndrome
29. _____ genito-urinary infections
30. _____ incompetent cervix
31. _____ alcohol abuse
32. _____ substance abuse
33. _____ placenta previa
34. _____ postmaturity
35. _____ threatened abortion
36. _____ tobacco use
37. _____ second or third trimester bleeding

_____ TOTAL Prenatal Risk
